# Updates in Laboratory Identification of Invasive Fungal Infection in Neonates

**DOI:** 10.3390/microorganisms11041001

**Published:** 2023-04-12

**Authors:** Binghong He, Qiong Yang

**Affiliations:** Beijing Key Laboratory of Gene Resource and Molecular Development, College of Life Sciences, Beijing Normal University, Beijing 100875, China; hehome1996@163.com

**Keywords:** invasive fungal infection, neonate, PCR, cationic conjugated polymer, CCP-FRET system

## Abstract

Invasive fungal infection (IFI) in immunocompromised neonates is significantly associated with high morbidity and mortality and has become the third most common infection in Neonatal Intensive Care Units. The early diagnosis of IFI for neonatal patients is difficult because of the lack of specific symptoms. The traditional blood culture remains the gold standard in clinical diagnosis for neonatal patients but it requires a long duration, which delays treatment initiation. Detections of fungal cell-wall components are developed for early diagnosis but the diagnostic accuracy in neonates needs to be improved. PCR-based laboratory methods, such as real-time PCR, droplet digital PCR, and the cationic conjugated polymer fluorescence resonance energy transfer (CCP-FRET) system, distinguish the infected fungal species by their specific nucleic acids and show a high sensitivity and specificity. Particularly, the CCP-FRET system, which contains a cationic conjugated polymer (CCP) fluorescent probe and pathogen-specific DNA labeled with fluorescent dyes, could identify multiple infections simultaneously. In the CCP-FRET system, the CCP and fungal DNA fragments can self-assemble into a complex with an electrostatic interaction and the CCP triggers the FRET effect under ultraviolet light to make the infection visible. Here, we summarize the recent laboratory methods for neonatal IFI identification and provide a new perspective for early clinical fungal diagnosis.

## 1. Introduction

Invasive fungal infection (IFI) is the most frequent infection in the Neonatal Intensive Care Unit (NICU) and one of the most serious complications in immunocompromised patients [1]. IFI can lead to tissue damage, organ dysfunction, and an inflammatory response, and most neonatal patients with IFI have atypical clinical manifestations [2]. The morbidity and mortality rate of IFI is extremely high, especially for premature infants with small gestational ages and low body weights [3,4,5]. The epidemiological studies on IFI in NICUs in China demonstrated the total IFI incidence rate of 42,187 neonates admitted to the hospital’s NICU was 2.42/1000 (102/42,187) during the five years from 2009 to 2014. Among them, 73.5% (75/102) of IFI patients were premature, 75.5% (77/102) were low-birth-weight neonates, and 8 of them died in hospital (7.8%), of which 7 were premature babies [6]. The data from the UK neonatal infections surveillance network from 2004 to 2010, through the standardization of the blood/cerebrospinal fluid (CSF) culture detection method of 14 neonatal units for monitoring, showed that the overall incidence rate of IFI in neonates admitted to the hospital was 2.4/1000, and the incidence rate of IFI in extremely low weight newborns was 18.8/1000 [4]. IFI is mainly caused by *Candida*, among which the main pathogens are *Candida albicans* (51.5%) and *Candida parapsilosis* (24.3%) [6]. In the UK, most IFIs are caused by *C. albicans* (69%) and *C. parapsilosis* (20%) [4]. In the United States, between 2009 and 2015, *C. albicans* infections accounted for 67% of neonatal candidiasis [5]. A survey concerning the period 2005–2010 carried out in the NICUs of Greater Lisbon indicated that the fatality rate of IFI was 11.4% [7]. A nationwide retrospective survey conducted in Japan from 2014 to 2015 showed that the mortality rate was 17.4% among the neonatal IFI patients [8]. Although the prophylactic antifungal agent treatment improves the IFI incidence of the infected infants [9], resistance to antifungal agents in surviving neonates calls for accurate diagnosis [10]. Appropriate and rapid diagnosis methods are needed to improve the recognition and administration of neonatal IFI.

As a gold standard, a blood culture is still the main clinical standardization for IFI diagnosis, but it is confined by being time-consuming and having a low sensitivity [11]. Meanwhile, contamination appeared frequently during blood cultures [12]. Thus, other laboratory methods, such as serum biomarker detection and fungal DNA detection have been developed, which speed up the identification of IFI. 1, 3-β-d-glucan (BDG) is a cell-wall constituent of *Candida* spp. and *Aspergillus*. The detection of BDG has been evaluated as a serum biomarker for IFI [13]. Serum biomarker detection shortens the diagnosis time and shows a higher sensitivity, but is challenging with indeterminate cutoff values in neonatal patients [14]. Fungus-specific PCR, figuring out infected pathogens by the specific nucleic acid fragment, is superior in accuracy and benefit to deep-seated infection [15]. Real-time PCR is a successively established PCR-based laboratory diagnosis method that improves the lowest detected amount [16]. Droplet digital PCR (ddPCR) improves the detection minimum. Furthermore, the cationic conjugated polymer fluorescence resonance energy transfer (CCP-FRET) system [17], relying on the PCR-based method, can identify three common pathogens separately or combinedly. *Candida albicans* (*C. albicans*), *Klebsiella pneumoniae* (*K. pneumoniae*), and *Cryptococcus neoformans* (*C. neoformans*) can be figured out simultaneously via different colors. A single color or overlay colors can be observed under UV light initiation in this system. In this review, laboratory identifications of neonatal IFI are summarized. Particularly, the CCP-FRET system, which can rapidly, accurately, and visibly identify multiple fungal infections, is a promising diagnostic method in NICUs.

## 2. Blood Culture

The blood culture is the gold standard for diagnosing bloodstream infection [18]. However, the positive rate of blood cultures is not high, and a significant proportion of patients with bloodstream infections have negative blood culture results [19,20]. The use of antibiotics before blood collection and the slow growth and high nutrient requirements of the pathogens, as well as the infection of some intracellular parasitic bacteria, can lead to negative blood culture results [19]. Sufficient blood samples for blood cultures are crucial for the detection of pathogens of bloodstream infection. The larger the blood collection volume, the higher the positive rate of blood cultures and the higher the diagnostic rate of bloodstream infection. The recommended blood collection volume is 8 ~ 10 mL per sample [21], but it is often difficult to achieve in the clinical operation of infants. Treatment of candidiasis is often delayed due to the <50% sensitivity of blood cultures to blood-borne disseminated candidiasis and the slow growth of candidiasis. The detection limit of the initial positive *Candida* blood culture in neonatal patients is ≤1 CFU/mL [22]. Indeed, the sensitivity of a blood culture is around 40–60% and could not indicate deep-seated infections [23]. Although increasing cultured blood volume promotes sensitivity, it is not suitable for newborns in poor conditions [24]. In addition, the blood culture has a major limitation, which is that it takes a long time to obtain cultures [25]. It usually takes 1 to 3 days to obtain positive cultures and further identification takes 1 to 2 days, which significantly delays the initiation of antifungal therapy and often misses the optimal time for antifungal therapy, leading to increased mortality in IFI patients. Moreover, Morrell M et al. revealed that antifungal therapy 12 h after the first positive blood culture sample was an independent determinant of hospital mortality (odds ratio (OR) = 2.09; *p* ≤ 0.018) [26]. Notably, some new strategies that are based on blood cultures, such as Peptide Nucleic Acid Fluorescent In situ Hybridization (PNA-FISH), allow a complete rapid detection of different *Candida* species [27]. For these reasons, blood cultures have been associated with biomarkers, imaging, and other clinical prediction methods to promote the diagnosis rate [28,29,30].

## 3. 1,3-Beta-d-Glucan (BDG)

BDG is an important fungal cell-wall component that is most frequently used to identify invasive candidiasis (IC), such as *Aspergillus* and *Candida* species [31]. When fungi enter the blood or deep tissue, BDG can be released after phagocytosis and digestion. The detection of serum BDG has a good diagnostic efficacy in the diagnosis of IFI in adult tumor patients or ICU patients, but its application in the diagnosis of IFI in children, especially neonates, is rare. The Fungitell test, a commercial method for BDG, was approved by the Food and Drug Administration (FDA) in 2004 and has been validated in adult IFI [32] and pediatric IFI [33]. However, the data on BDG in neonatal IFI are scarce, and the BDG assay shows a poor sensitivity and a high false-positive rate [34]. Although the BDG level in suspected IC-infective neonates was higher than that of the noninfected group (364 pg/mL vs. 89 pg/mL; *p* < 0.001), the optimal BDG cutoff in the assay was 125 pg/mL, which was much higher than the adult recommended value (80 pg/mL) [35]. The cutoff values significantly influenced the specificity of the neonatal IFI, indicating the rationale of the cutoff values remains negotiated. When the recommended cutoff was increased from 80 pg/mL to 174 pg/mL, the specificity elevated from 51.9% to 77.8% [36]. A study aiming to characterize the baseline values and dynamics of BDG for neonates showed that the median BDG value of the enrolled neonates was 59 pg/mL (IQR 30-148), and the mean value was 119 pg/mL (SD ± 154). However, the Fungitell assay used for testing has a recommended cutoff value of 80 pg/mL, indicating that 42.1% of samples were higher than the cutoff values. However, none of the enrolled patients were diagnosed with IFI [37]. This was expected for the uncertain cutoff value, and some medical sources, such as dialysis membranes, filters made from cellulose, and cotton gauze, could lead to a false-positive assay in BDG [38].

## 4. Galactomannan (GM)

GM is a specific antigenic component of *Aspergillus* [39] that is released into a serum when *Aspergillus* colonizes. The GM antigen detection test was first introduced in 1995 and received FDA approval in 2003 [40]. A meta-analysis of serum GM test performance in 178 patients with proven or probable invasive aspergillosis (IA) showed the sensitivity and specificity were 85% and 88%, respectively, with a cutoff value of 0.5 [41]. A single GM performed in 6 premature neonates indicated a high false-positive value of 83.3% [42]. Moreover, the sensitivity and positive predictive value were reduced to 0% when the GM assay was conducted alone after the patients received antifungal prophylaxis [43]. Above all, GM testing is characterized by a low sensitivity in pediatric patients with primary immunodeficiencies [44] and was not suitable for the NICU IFI infectors.

## 5. Multiple Nested PCR

Multiple nested PCR can detect multiple fungi in the same reaction tube within 24 h, and this determination can identify *Candida* species that are 100% in line with blood cultures [45,46]. The advantage of nested PCR is that the use of inner and outer nested primers greatly improves the specificity and sensitivity of the detection and realizes multiple detections. However, the low yield of DNA in blood and false positivity caused by fungal contamination or nonspecific primers limit the widespread uptake of PCR diagnosis. The lack of a standardized methodology is also a concern. Therefore, it is not suitable to routinely use PCR for the diagnosis of IC in neonates [47,48]. Most notably, the combination of PCR and the detection of BDG can effectively detect deep-seated candidiasis, which is often impossible to detect by blood culture method. Nguyen et al. found that, for the sensitivity of patients with deep-seated *C. albicans* disease, PCR (88%) > the detection of β-glucan (62%) > blood culture (17%) [49]. The combination of PCR and nanoparticle-based hybridization (T2 magnetic resonance) rapidly (<5 h), accurately, and reproducibly detects 1–3 CFU of *C. albicans*, *C. tropicalis*, *C. glabrata*, *C. krusei*, and *C. parapsilosis* per milliliter of spiked blood or in patients with a low incidence of IC.

## 6. Real-Time PCR

Currently, DNA barcoding is utilized to identify different species of fungi, which are mainly composed of species-specific and highly conserved sequences [50]. For instance, the internal transcriptional interval (ITS) of ribosomal DNA is considered the universal barcode of fungi by default [50,51]. In addition to the ITS rRNA operon, D1-D2 regions with high variable sequences of the 18S rRNA gene and 28S rRNA gene are also often used as target genes for PCR detection [17]. Recent studies have shown that sequences other than D1-D2 regions in the 28S rRNA gene can also be used for PCR detection of broad-spectrum fungi. Based on PCR, real-time PCR was developed for laboratory IFI diagnosis to shorten detection time [26,52]. The minimum sensitivity of real-time PCR was 30 CFU/mL for *Candida albicans*, *Candida tropicalis*, *Candida krusei*, and *Aspergillus fumigatus* in the early detection and identification of neonatal sepsis [53]. A multiple real-time PCR conducted in 208 suspected sepsis neonatal specimens demonstrated that only 45% of infected samples were identified [54]. However, another study indicated that a real-time-PCR-based assay showed that it is superior to the BDG method. The detection of very-low-birth-weight preterm neonatal patients’ infection showed the sensitivity and specificity of the real-time PCR were 87.5% and 81.6%, respectively, while for the BDG method they were 75.0% and 64.6% [14]. A new panfungal real-time PCR assay was developed using an intercalating dye and sequence-specific probes. In addition, a melting curve analysis was also performed following DNA amplification. The technique was validated in 60 clinical samples from patients with proven and probable IFI using 11 different fungal species. The results demonstrated a high reproducibility (5% CV; r > 0.95) and specificity (100%). This method’s overall sensitivity was 83.3%, with the group of fungi involved in the infection detected in 77.8% of positive samples with IFIs detected by molecular beacon probes. Furthermore, in 67.8% of these “probe-positive” results, sequencing was avoided, allowing a positive result to be reported within 24 h. In this way, panfungal real-time PCR is quick, sensitive, and specific, and it has the potential to improve the early detection of IFIs [55].

## 7. Droplet Digital PCR (ddPCR)

Droplet digital PCR (ddPCR) is a novel way to improve regular PCR. In a digital PCR, the sample could be compartmentalized into several small bioreactors, each of which has either zero or one or two (or three, four, etc.) copies of the target nucleic acids. Each droplet is a separate reaction and has a particular encapsulated area that prevents cross-contamination. A small percentage of the droplets contain one or fewer copies of the DNA template and are then clonally amplified in each microdroplet. Following standard PCR amplification, concentrations are calculated using the Poisson distribution’s share of nonfluorescent segments [56]. In addition, ddPCR provides many benefits compared to conventional qPCR. Amplicons are calculated at the end of each step in a qPCR method using DNA-binding dyes. The fluorescent dyes attached to dsDNA have an intensity proportional to the PCR amplicons. For the specific absolute quantification of viral nucleic acid by qPCR, a standard curve is required. However, ddPCR does not require a calibration curve. ddPCR uses the same primers, probes, Taq polymerase, and reagents as conventional PCR to amplify the target DNA fragment, but its sensitivity and repeatability are more significant.

Droplet-based digital PCR is a new technology for neonatal IFI in which the DNA/RNA of the pathogen is encapsulated randomly inside the microdroplets and the PCR amplification is initiated in each of these microdroplets. Only a small percentage of the PCR reactions contain the objective DNA or RNA that can indicate the infection [56]. After selecting the highly conserved 18S rRNA gene as the detective sequence of fungi, the ddPCR detection system was established with primer design and system optimization. The sensitivity and the specificity of ddPCR for detecting IFI-positive neonates were 86% (19/22) and 97% (59/61), respectively, [57] and the minimum of detection was 3.2 copies/μL [58].

## 8. CCP-FRET System

Based on the nucleic acid probe molecule, we have established sensitive and specific pathogen experimental detection methods in vitro. The probe-molecule-mediated fluorescence resonance energy transfer (FRET) technique might provide a new diagnostic method for molecular diagnostics of IFI in neonatal patients [17]. The CCP is one kind of macromolecule with a skeleton made up of alternating saturated and unsaturated carbon links [59], which could absorb ultraviolet (UV) light and transfer energy through its π-π* transition to the combined molecules via the FRET effect. Given the fluorescence signal amplification effect, the CCP-FRET system, which is comprised of a water-soluble conjugated polymer and a small molecule fluorescence dye, was established to be a simple and efficient approach for detecting particular DNA fragments [60]. When the CCP and the DNA labeled with a small molecule dye were electrostatically bonded, the effective FRET occurred and considerably boosted the dye’s fluorescence signal to improve detection sensitivity [61].

The CCP-FRET system has been widely used for highly sensitive DNA detection. For example, Duan et al. achieved homogeneous single nucleotide polymorphism (SNP) detection on a p53 mutation through the allele-specific extension [62]. The dGTP-FL might incorporate into the polymorphic site when the target/probe pair is complementary. Feng et al. used the CCP-FRET to differentiate the DNA methylation for the highly selective single-base extension (SBE) on colon cancer [63]. Specific methylation primers were designed to identify the colon cancer cells and the FRET effect between the DNA and CCP was triggered under UV. The superior fluorescence signal amplification via the CCP-FRET system achieved specific gene mutation detection [64,65] to identify two or three kinds of SNPs. Recently, Shen et al. established a rapid detection of multiple genetic loci by CCP-FRET and 10 genetic loci were successfully identified to benefit the treatment of hypertension [66].

**Figure 1:**
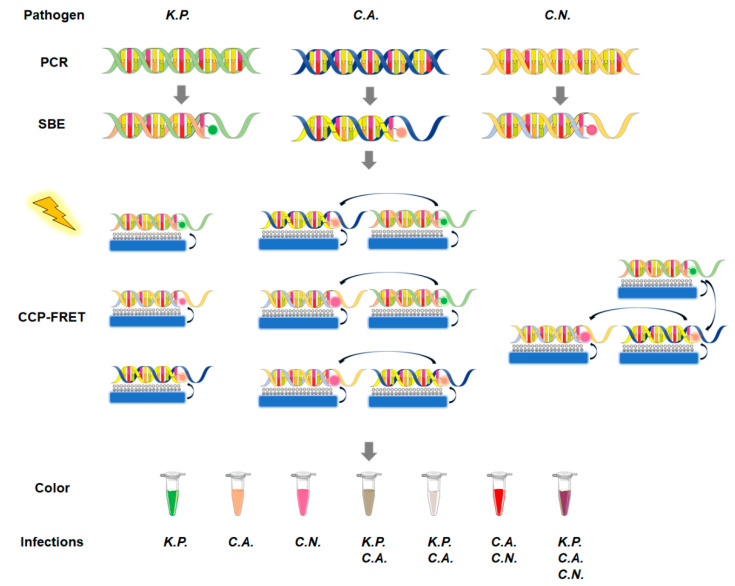
Schematic illustration of the CCP-FRET system. The ITS DNA of the three pathogens is enriched through regular PCR and then the dye-ddNTPs are connected to the specific DNA fragments by a single-base extension (SBE) reaction. The CCP-FRET reaction is triggered by 380 nm UV light and CCP acts as an energy donor to activate the pathogen-specific DNA labeled with fluorescent dyes. In the system, different colors can be observed for different combinations of infection in the reaction tubes. *K.P.* represents *K. pneumoniae*, *C.A.* represents *C. albicans*, and *C.N.* represents *C. neoformans*.

Therefore, taking advantage of this feature, different pathogens could be identified with their pathogen-specific DNA fragments. The CCP-FRET system was developed for neonatal IFI detection [17] (Figure 1). Unique nucleic acid fragments of the pathogens were amplified by PCR, and then the SBE reaction was initiated with pathogen-specific primers by which the dye-ddNTPs were incorporated into the 3’ ends of the specific primers, respectively. When the reaction system was excited with 380 nm UV light, a pathogen-strain-specific FRET signal was generated between the CCP and the fluorescent dye molecules. Since one dye-labeled DNA fragment could represent one kind of pathogen, different species could be determined with the different dyes. Given the excitation spectrum of the dyes, three dye-ddNTPs that have overlapping excitation spectra were used one by one in the systems to figure out three different pathogens (*K. pneumoniae*, *C. albicans*, *C. neoformans*). In this system, three single pathogen strains and their combinations could be differentiated using FRET emission spectra. After the extraction of the pathogen DNA, a PCR reaction was undergone for the amplification of the specific 5.8S or 16S rDNA fragments of these three candidates. Moreover, in the SBE reaction, the specific DNA fragments could connect to their complementary primer, where the dye-ddNTPs were respectively incorporated into the specific primer’s 3′-terminus. Since the complementary base varies from the DNA fragments, the different pathogens would bind different dye-ddNTPs. The CCP-FRET effect would be triggered under UV light in a solvent containing CCP molecule and SBE products. CCP-FRET acts as the signal amplifier of the dye-ddNTPs, displaying different colors for the existence of different pathogens. In this way, the results could be observed with the naked eye, realizing a fast and visible detection. Such a system realizes the identification of *K. pneumoniae* (*K.P.*), *C. albicans* (*C.A.*), and *C. neoformans* (*C.N.*). According to the experimental design, specific DNA fragments of K.P. would connect to FL-ddUTPs that could be visible with a green color under the trigger of UV light. As for *C.A.*, ROX-ddATP is incorporated into the primer’s 3′-terminal OH of the specific DNA and the orange color could be observed when it was triggered by UV light. In addition, *C.N.* could be observed with a pink color for Cy5-ddGTPs incorporating into its primer’s 3′-terminal OH. Once more than one pathogen was contained in the detective system, these dye-ddNTPs could be initiated simultaneously and their colors overlapped. At this time, different colors exhibit different infections. The relative colors in this assay were as follows (Figure 1).

Since the emission wavelength for dye-ddNTPs was ROX at 605 nm, Cy5 at 668 nm, or FL at 517 nm, the different colors could be triggered in the solutions containing IFI candidates. For *C.A.*, an efficient FRET from CCP to ROX led to a CCP emission and an increase in the ROX emission peak at 605 nm, which exhibited a violet color in the reaction solution. For *C.N.*, the Cy5 at 668 nm was emitted, and the solution showed an orchid color. For K.P., the FL at 517 nm was emitted and showed a light sky-blue color. For *C.N.* and *C.A.* combination, the ROX and Cy5 were be emitted at the same time, where the reaction system showed a dim gray color. Similarly, for other types of pathogen strain combinations, the corresponding ROX, Cy5, and FL’s emissions were be emitted and the reaction systems gave specific fluorescent colors. Since the CCP as the donor could transfer the energy to the other dyes, the different pathogens’ DNA fragments containing dye-ddNTPs could be triggered and exhibited different colors in a compact CCP self-assembly system.

As rapid as the CCP-FRET system is, only 3 h are needed to finish the identification process from DNA extraction to the final confirmation of pathogenic infections. Moreover it is worth mentioning that the detection limit of the CCP-FRET system was 0.03125 ng DNA, which is one-tenth that of real-time PCR [17]. Furthermore, high specificity is also guaranteed in CCP-FRET, meaning that other nontarget strains would not trigger this system. A double-blind test of clinical specimens demonstrated that only 200 µL blood samples were needed to identify the pathogens by the CCP-FRET system. The specificity of CCP-FRET in clinical specimens was up to 100% and the sensitivity for multiple detections in one reaction tube was 100% for *K.P.* and *C.A.* infections, whose results were consistent with the clinical blood culture.

Below, there is an overview of the laboratory identification of IFI in neonates to make a clear comparation of the pros and cons of these methods (Table 1).

## 9. Conclusions

The development of diagnostic methods in the NICU is pivotal and minimizes the mortality of newborns. Different methods own their cons and pros but all contribute to identify infected pathogens and alleviate the discomfort of babies. Methods with a higher sensitivity and specificity are needed to achieve effective and accurate diagnosis and treatment. Although empirical treatment has improved many sufferings, accurate and early identification methods are still necessary to improve the overuse of antibiotics. Improving detection efficiency can reduce the economic burden on the one hand, and buy more treatment time on the other hand. Neonates in the NICU, especially low-birth-weight infants, are very vulnerable, and using less blood collection to achieve more accurate results is what we are working towards.

Different diagnosis methods for IFI are urgently needed to reduce morbidity and fatality at an earlier stage for NICU patients. The blood fungal culture is still preserved in traditional ways for providing morphological identification. However, the long time duration and low sensitivity prevent it from becoming the main rapid detection method for IFI. Fungal cell-wall polysaccharide identification and specific fungal DNA detection shorten the identification time and identify the pathogens from their specific components. Though the BDG assay is mature in adult IFI detection, the cutoff value of BDG for neonates remains uncertain. GM testing for neonates showed a low sensitivity, which limited its application. PCR-based methods have been rapidly developed and optimized to adapt to the needs of neonatal IFI diagnosis, showing superior advantages in identifying different pathogens in deep infection. Multiple nested PCR achieves multiple identifications in the same reaction but it lacks a standardized methodology. Real-time PCR and ddPCR improve the sensitivity and specificity of the IFI. However, their sensitivity and specificity are still nonuniform. In addition to these two kinds of PCR-based methods, every reaction could only detect one kind of pathogen infection. The CCP-FRET system makes the pathogen infection visible, in a timely and convenient method, paving a new way for the development of the early detection of IFI. Meanwhile, CCP-FRET can identify up to three kinds of pathogens in one reaction, saving time and blood volume. It is a simple and visible method that should be promoted in areas with poverty.

Since the CCP-FRET system is a rapid, sensitive, and specific method for possible IFI identification, it is recommended that hospitals should establish their own CCP-FRET system with their most frequent pathogens. When a neonatal patient is considered under infection, instead of the regular diagnosis methods, CCP-FRET could be performed with a 200 µL blood sample. The CCP-FRET results could be obtained within 3 h and provide the clinicians with some suggestions for treatment. In summary, an earlier and more accurate diagnosis of IFI in neonatal patients is expected for the better identification of the devastating infection in neonatal patients at risk for IFI.

## Figures and Tables

**Table 1:** Overview of the Laboratory Identification of Invasive Fungal Infection in Neonates.

Methodology	Advantages	Disadvantages	References
Blood culture	Detection of fungal pathogen	Long turn-around time Low sensitivity	[18,19,20]
BDG	Detection of relevant infection Time-saving	Nonspecific Poor sensitivity High false-positivity	[31,34]
Galactomannan	Detection of Aspergillus infection Noninvasive Time-saving	Low sensitivity High false-positivity	[41,42]
Multiple nested PCR	Detection of multiple fungi simultaneously High sensitivity Time-saving	High false-positivity Lack of a standardized methodology	[47,48]
Real-time PCR	High specificity High sensitivity Time-saving	Contamination Lack of a standardized methodology	[14,53,54]
ddPCR	High sensitivity Time-saving	Lack of a standardized methodology	[57]
CCP-FRET system	High sensitivity Multiple fungi detection High specificity Time-saving	Careful selection of primers and optimization	[17]

## Data Availability

Data sharing not applicable.
